# A double-blind, randomised, placebo-controlled trial of *Ganoderma lucidum* for the treatment of cardiovascular risk factors of metabolic syndrome

**DOI:** 10.1038/srep29540

**Published:** 2016-08-11

**Authors:** Nerida L. Klupp, Hosen Kiat, Alan Bensoussan, Genevieve Z. Steiner, Dennis H. Chang

**Affiliations:** 1 The National Institute of Complementary Medicine, School of Science and Health, Western Sydney University, Penrith NSW, Australia; 2 Faculty of Medicine, University of New South Wales, Kensington NSW, Australia; 3 School of Medicine, Western Sydney University, Penrith NSW, Australia; 4 Faculty of Medicine and Health Sciences, Macquarie University NSW, Australia

## Abstract

This study aimed to evaluate the efficacy and safety of *Ganoderma lucidum* for the treatment of hyperglycaemia and other cardiovascular risk components of metabolic syndrome using a prospective, double-blind, randomised, placebo-controlled trial. Eighty-four participants with type 2 diabetes mellitus and metabolic syndrome were randomised to one of three intervention groups: *Ganoderma lucidum*, *Ganoderma lucidum* with *Cordyceps sinensis,* or placebo. The dosage was 3 g/day of *Ganoderma lucidum,* with or without *Cordyceps sinensis,* for 16 weeks. The primary outcome measure was blood glucose (glycosylated haemoglobin [HbA1c] and fasting plasma glucose [FPG]); a number of secondary outcome measures were also tested. Data from the two intervention groups were combined. The combined intervention had no effect on any of the primary (baseline-adjusted difference in means: HbA1c = 0.13%, 95% CI [−0.35, 0.60], *p* = 0.60; FPG = 0.03 mmol/L, 95% CI [−0.90, 0.96], *p* = 0.95) or secondary outcome measures over the course of the 16-week trial, and no overall increased risk of adverse events with either active treatment. Evidence from this randomised clinical trial does not support the use of *Ganoderma lucidum* for treatment of cardiovascular risk factors in people with diabetes mellitus or metabolic syndrome. This Clinical Trial was registered with the Australian New Zealand Clinical Trials Registry on November 23, 2006. Trial ID: ACTRN12606000485538 and can be accessed here: https://www.anzctr.org.au/Trial/Registration/TrialReview.aspx?id=81705.

One of the leading causes of death worldwide is cardiovascular disease^1^. This term most commonly refers to the chronic diseases caused by atherosclerosis, and most notably includes coronary heart disease and cerebrovascular disease. These conditions result in a significant burden on morbidity, quality of life, economic status and mortality for individuals and populations. Although the risk factors for cardiovascular disease are numerous and varied, the most important pharmacologically modifiable risk factors are high blood glucose, high blood pressure, an abnormal lipid profile, and obesity^1^,^2^,^3^,^4^,^5^.

The above risk factors are not independent of one another^6^. It has been established that hyperglycaemia, hypertension, high triglycerides, low levels of high-density lipoprotein (HDL) cholesterol, and obesity have a complex metabolic relationship with insulin resistance, cardiovascular disease, and with each other^7^; this combination of risk factors has been described as metabolic syndrome. Recognising this multifactorial nature of risk has had significant implications for the effective management of cardiovascular disease in individuals and populations.

Modifiable risk factors, including those of metabolic syndrome, can be effectively treated with lifestyle modifications^2^,^3^,^4^,^8^,^9^. Unfortunately, it is rare for these effects to be successfully sustained in the long term and consequently, pharmaceutical interventions are required for the majority of individuals. There is no single medication for treatment of metabolic syndrome due to its multifactorial components. Furthermore, medications that treat cardiovascular risk factors might be associated with contraindications, adverse events, insufficient efficacy, polypharmacy concerns and increased risk for co-existing factors.

*Ganoderma lucidum,* a traditional Chinese medicinal mushroom also called Ling Zhi or Reishi, has been used in China to promote health, prolong life and prevent and cure systemic diseases for more than 2,000 years. In recent times several small studies suggest *Ganoderma lucidum* is well tolerated and might improve multiple cardiovascular risk factors including blood pressure, blood glucose, triglycerides and cholesterol profiles^10^,^11^,^12^.

*In vitro* and diabetic rodent studies suggest that *Ganoderma lucidum* can improve blood glucose and serum insulin levels, whilst protecting pancreatic islets from free radical damage^13^,^14^,^15^,^16^,^17^. Other animal work has demonstrated reduced plasma total and low-density lipoprotein (LDL) cholesterol, triglycerides, and phospholipid levels, and increased HDL cholesterol^18^, with some studies reporting unchanged triglycerides^19^ and decreased HDL cholesterol^10^. Some human studies with intervention lengths of 4–12 weeks in patients with type 2 diabetes mellitus or hypertension have reported improvements in glycosylated haemoglobin (HbA1c), fasting plasma glucose (FPG), postprandial glucose, insulin, and C-peptide^20^, total cholesterol^21^, LDL cholesterol^22^, blood pressure^21^ for individuals with essential^11^ or nonresponsive^23^ hypertension, whilst other studies have reported no changes in glucose parameters^24^,^25^, and unchanged LDL cholesterol^25^. However, for all these clinical trials reporting positive results, the reporting of methods was poor and unclear, with a high potential for bias (for a systematic review, see Klupp *et al*.^26^).

*Ganoderma lucidum* is sometimes combined with a second mushroom extract, *Cordyceps sinensis*, which is thought to increase the potency of *Ganoderma lucidum*. *In vitro*, animal, and small human studies have shown *Cordyceps sinensis* to be antihyperglycemic, antisenescent, anti-atherosclerotic, antihypertensive, antioxidant, antitoxic, and renal and hepatic protective^27^,^28^,^29^,^30^,^31^,^32^,^33^,^34^,^35^,^36^, and that it can improve total, HDL, LDL, and very-LDL (vLDL) cholesterol^37^,^38^,^39^, and lower triglycerides^30^.

Both *Ganoderma lucidum* and *Cordyceps sinensis* are listed as the safest drug class (Class 1 Drug) in the *American Herbal Products Association Botanical Safety Handbook*^40^, with no known herb-drug interactions. There are no scientific data on a safe and effective dosage of *Ganoderma lucidum*. Recommended amounts derived from historic Chinese medicine tradition, expert opinion and marketing trends vary between 1.5 g and 9 g of dried extract per day^41^,^42^. Most human clinical trials investigating *Ganoderma lucidum*^20^,^21^,^22^,^43^,^44^,^45^,^46^,^47^,^48^,^49^,^50^,^51^ or *Cordyceps sinensis*^38^ have included safety haematological and biochemical (including hepatic and renal) biomarkers, and reported no pathological abnormalities, and no moderate, serious or severe adverse events. Mild herbal sensitivity side effects were noted for some participants after *Ganoderma lucidum* including dry mouth, sore throat and nausea, with a smaller proportion of participants reporting vomiting, headache, diarrhoea, dizziness, insomnia and shortness of breath^20^,^21^,^22^,^43^,^44^,^45^,^46^,^47^,^48^,^49^,^50^,^51^. It should be noted that there has been one report of a severe adverse event (hepatotoxicity) after 1 month supplementation with *Ganoderma lucidum*^52^, however this was thought to be due to the excipient ingredients.

The use of complementary and alternative medicines is increasing worldwide. In Australia, the most recent national consumer survey found 67% of the population reported using a complementary medicine in the previous 12 months^53^. Some of the reasons provided for using complementary medicines are perceptions that they are safer, more ‘natural’ and more holistic than Western medicines^54^. There is also evidence that Australians have less concerns about safety, efficacy and medication interactions for complementary medicine than for other pharmaceuticals, and that up to 50% of consumers do not discuss their complementary medicine use with their regular doctor^54^,^55^,^56^. This, taken together with estimates that Australians are spending up to four times more money on complementary medicines than pharmaceuticals^57^, emphasises the importance of evaluating the efficacy and safety of such medicines, including *Ganoderma lucidum*. The purpose of this clinical trial was to address this important issue, especially given that previous clinical trial results on *Ganoderma lucidum* might have high potential for bias.

The aim of this prospective randomised controlled trial was to evaluate the efficacy and safety of *Ganoderma lucidum,* with or without *Cordyceps sinensis,* for treatment of hyperglycaemia and cardiovascular risk factors in persons with metabolic syndrome. As metabolic syndrome has five important components and no validated summary value, hyperglycaemia, the component with the most compelling evidence base for the benefit of *Ganoderma lucidum*, was selected for the primary research question. It was hypothesised that *Ganoderma lucidum* (with or without *Cordyceps sinensis*) would reduce hyperglycaemia (measured by HbA1c and FPG), and would be well tolerated by participants.

## Methods

### Trial Design

This study was conducted as a multiple-blinded, randomised, placebo-controlled, parallel design clinical trial. No changes were made to this trial design after recruitment had commenced. This clinical trial received ethics approval from the Western Sydney University and was registered with the Australasian Clinical Trials Registry (ACTRN012606000485538). All experiments were carried out in accordance with the approved ethical guidelines. Organisations who contributed funds to this collaborative trial are Allife Australia Pty Ltd, Western Sydney University, Cardiac Health Institute, and Sydney Adventist Hospital. This was an investigator-led trial, and these organisations had no involvement or influence on study design, conduct, analysis or reporting.

### Participants and Setting

Participants were recruited through media advertisements and general practitioners. Inclusion criteria were for participants to be aged 18 years and older, have high fasting serum glucose ≥6.1 mmol/L, and at least two other National Cholesterol Education Program-Adult Treatment Panel III (NCEP-ATP III) diagnostic criteria for metabolic syndrome (fasting serum triglycerides ≥1.7 mmol/L, blood pressure ≥130/85 mmHg, waist circumference >102 or 88 cm and HDL cholesterol <1 or 1.3 mmol/L for men and women, respectively). All participants had a diagnosis of type 2 diabetes mellitus. Exclusion criteria were: known mushroom allergy, pregnancy or breastfeeding, administration of *Ganoderma lucidum* or *Cordyceps sinensis* or insulin treatment in the past 12 months, serious psychiatric or medical conditions (e.g., malignancy, heart disease, renal impairment, and hepatitis), history of abnormal haemorrhaging, use of an anticoagulant or a hypoglycaemic event in the past 3 months. Participants were permitted to continue taking any current medications.

Initially, participants were screened via telephone or e-mail before being selected to attend a more thorough screening interview where written informed consent was provided, a complete medical history and pathology sample was taken, and a physical examination was conducted. Participants then attended monthly morning appointments to have their pathology samples collected, medications dispensed, and to return any unused medications. All testing sessions took place at the Cardiac Health Institute (CHI), Sydney, Australia. A copy of the trial protocol is available here: https://www.anzctr.org.au/Trial/Registration/TrialReview.aspx?id=81705.

### Interventions

Participants took one of three interventions: *Ganoderma lucidum, Ganoderma lucidum* with *Cordyceps sinensis*, or placebo that were manufactured and packaged according to Australian Good Manufacturing Practice and Therapeutic Goods Administration (TGA) standards. The compositions of these interventions with respect to their daily dosage (mg) are detailed in Table 1. Based on recommended dosages from the literature^41^,^42^, all participants took eight capsules (3g of *Ganoderma lucidum,* with or without *Cordyceps sinensis*) per day (four in the morning and four at night), orally and with food, for a period of 16 weeks. An excipient of ascorbic acid, carob flour and caramel colour was used in all three capsules to ensure that they were indistinguishable in colour, smell, taste, size and weight.

### Randomisation and Blinding

Eligible participants were given the next participant project number that had previously been randomly allocated to one of the three trial arms using a standard computerised randomisation strategy (generated in Microsoft Excel by a research officer external to the research team), with blocking to create equal group numbers. The participants, therapists, assessors and data analysts had no knowledge of participant group allocations for the duration of the trial. All participants were involved with identical stages and activities of the clinical trial.

### Outcome Measures

A list of primary and secondary outcome measures, and the timing of their collection throughout the 16-week clinical trial, and subsequent follow up at 24 weeks, is outlined in Table 2.

### Sample Size and Statistical Analyses

For a three-arm trial a prospective sample size calculation using Friedman’s formula estimated that 168 participants (56 per group) would detect a 1% change in HbA1c (α = 0.05, *SD* = 1.4) at 90% power, representing a 12% improvement from a baseline measure of 8% HbA1c, and allowing for a total noncompliance/withdrawal rate of 20%. The smallest worthwhile clinical effect was also determined for the FPG co-primary outcome measure as 1.5 mmol/L, which would represent a 20% improvement from a baseline measure of 7.5 mmol/L. The sample size needed to detect this effect was much smaller (39 participants at 90% power, α = 0.05, *SD* = 1.0, allowing for 20% noncompliance). However, the anticipated sample size was not reached during the 2-year recruitment period. To increase statistical power, and because the two intervention groups contained the same quantity of the active constituent, the *Ganoderma lucidum* group and the *Ganoderma lucidum* with *Cordyceps sinensis* group were pooled to create a combined intervention group for all analyses.

Baseline differences on demographic and clinical characteristics were calculated using independent-samples *t*-tests and Chi-squared goodness of fit tests. To evaluate the efficacy of the combined intervention group against placebo, between-group differences in primary (HbA1c and FPG) and secondary outcome measures (the other four metabolic syndrome criteria, health-related quality of life, C-reactive protein, total and LDL cholesterol, and apolipoproteins A and B) at 16 weeks were evaluated using separate ANCOVAs, with baseline score as the covariate. A treatment effect size was calculated at the end point of the intervention, and if statistically significant (*p *< 0.05), the number needed to treat (NNT) was also calculated. The data of any participant who withdrew from the trial were handled according to the intention-to-treat last observation carried forward (LOCF) method in relation to their randomised group. As the study was underpowered for inferential testing of metabolic syndrome diagnostic criteria and safety variables, these were evaluated descriptively. There were no interim analyses.

## Results

**Figure 1:**
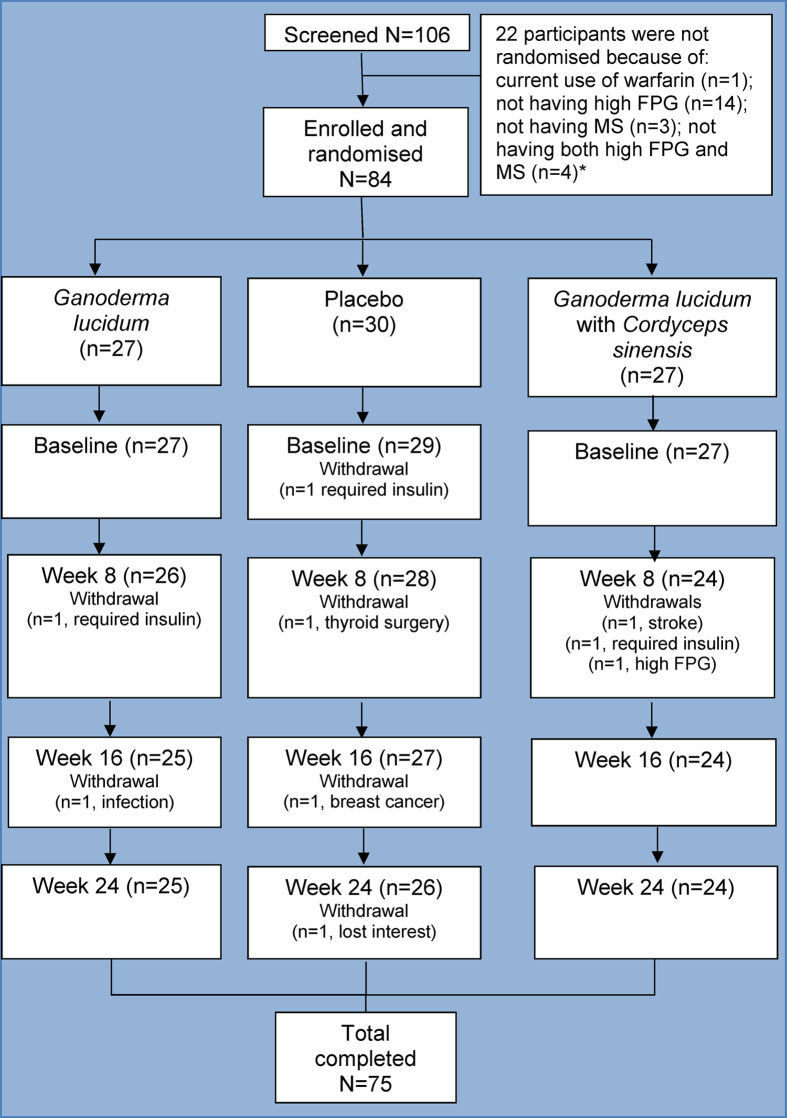
Participant flow.

Over the 2-year recruitment period, there were more than 1,000 initial participant screenings by phone and e-mail. Recruitment commenced January 25, 2007 and follow up of the final participant ceased on October 31, 2008. As shown in Fig. 1, from these initial enquiries, 106 persons were deemed suitable for in-person screening for inclusion in the clinical trial. Of these, 84 met the inclusion criteria. Nine persons withdrew from the clinical trial; reasons for withdrawal are presented in Supplementary Materials. Compliance was determined by number of missed doses of medication; each participant self-reported taking a minimum of 112 doses (896 capsules). Other than withdrawals and missed doses, there were no other observed deviations from protocol. Success of blinding was also checked at mid- (8 weeks) and end-points (16 weeks); at these key time points, for every one participant who guessed their group correctly, three participants did not, indicating successful blinding. Participant demographics at baseline are detailed in Table 3. There were no significant differences in baseline age, sex, country of birth, language, level of education, employment or carer status between groups, oral hypoglycaemic medication use, or any of the primary or secondary outcome measures at baseline between the two groups.

Table 4 shows the mean and *SD* for each primary and secondary outcome measure for placebo and combined intervention groups at baseline and endpoint (Week 16). The baseline-adjusted difference in means and 95% confidence interval, the test statistic and *p*-value are shown in the three right columns, respectively. After controlling for baseline differences, combined *Ganoderma lucidum* did not significantly affect any of the outcome measures (co-primary: HbA1c and FPG; and secondary: blood pressure, triglycerides, waist circumference, BMI, health-related quality of life, C-reactive protein, total, HDL and LDL cholesterol, and apolipoproteins A and B) compared to placebo at 16 weeks.

**Figure 2:**
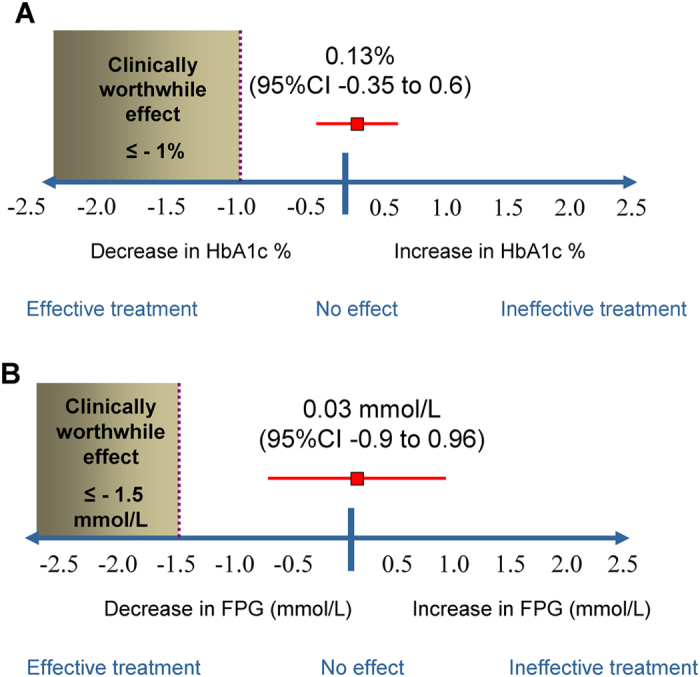
Clinical effect of combined *Ganoderma lucidum* against the smallest worthwhile clinical effect for HbA1c (panel A, upper) and FPG (panel B, lower).

Figure 2 is a tree plot illustrating the clinical effect of combined *Ganoderma lucidum* against the smallest worthwhile clinical effect for HbA1c (panel A, upper) and FPG (panel B, lower). The observed clinical effect sizes (0.13% increase in HbA1c and 0.03 mmol/L increase in FPG) did not overlap or approach the respective *a priori* worthwhile clinical effects of 1% and 1.5%. As a result, the NNTs were not calculated.

Common side effects did not cause a significant concern or discomfort to the participants. The most common adverse event for all groups was an infection or immune system condition, and these were reported most frequently by participants in the placebo group. The only two serious adverse events, which were reported to the TGA, were not attributed to the trial intervention, as determined by the clinical trial medical team and external medical personnel. The frequency of anticipated mild adverse events (side effects) and non-anticipated adverse events is summarised in Supplementary Materials.

## Discussion

This prospective, double-blind, randomised placebo-controlled trial did not demonstrate any significant beneficial effect of 16 weeks treatment with *Ganoderma lucidum* for hyperglycaemia and cardiovascular risk factors among individuals with type 2 diabetes mellitus and metabolic syndrome. As the anticipated sample size was not reached, and the two interventions groups contained the same quantity of the active constituent, the *Ganoderma lucidum* groups, with and without *Cordyceps sinensis*, were combined for all analyses.

Counter to previous human trials with less robust designs^11^,^20^,^21^,^22^,^23^, *Ganoderma lucidum* in our study did not reduce either of the co-primary outcome measures, HbA1c or FPG, during the 16-week treatment period. The treatment effect of *Ganoderma lucidum* on HbA1c and FPG was close to zero, with the direction of both effects favouring the control group; the magnitude of change was too negligible to suggest harm from the intervention. The magnitude of effects was also too small to measure the NNT or number needed to harm (NNH).

For all secondary outcomes, there were no statistically significant differences between the adjusted means of the combined *Ganoderma lucidum* group and the placebo group. There were also no clinically significant improvements as a result of *Ganoderma lucidum* treatment over 16 weeks. Any changes in secondary outcome measures were negligible. For example, mean arterial blood pressure decreased by 0.3 mmHg, triglycerides by 0.1 mmol/L, C-reactive protein by 0.7 mg/L, and HDL and BMI did not change. There was also no reduction in the number of participants who met the diagnostic criteria of metabolic syndrome in those taking *Ganoderma lucidum* compared with the placebo group. These findings are in line with some work^25^, but are the opposite of what smaller studies at high risk of bias have reported^11^,^21^,^22^.

The majority of adverse events were mild. Participants in the *Ganoderma lucidum*, *Ganoderma lucidum* with *Cordyceps sinensis*, and placebo groups each reported 52, 35, and 43 adverse events, respectively. The only two serious events (stroke and diagnosis of breast cancer) were not considered attributable to the trial intervention. Pathology biochemistry panels were also evaluated for safety of *Ganoderma lucidum* and were found to be similar between intervention and control groups.

This study aimed for contemporary best practice in clinical trial design, implementation, interpretation and reporting. Each stage was performed in accordance with international, national and institutional ethical and research guidelines. Assessment with the Jadad Scale^58^, PEDro Scale^59^, and Cochrane Risk of Bias Tool^60^ all indicated that this study was at low risk of bias due to randomisation, allocation concealment, blinding of participants, therapists and assessors; level of attrition, intention-to-treat analyses, reporting of between-group effect estimates for pre-specified outcomes, and conflict of interest.

Standards for best clinical practice are also client-centred. Clinical research can support this by ensuring efficacy studies include evaluations of safety and tolerability, reporting of clinical significance (rather than just statistical significance), and a participant perspective of the experience. In this trial, safety of *Ganoderma lucidum* was evaluated by frequency and severity of adverse events and atypical pathology results. The SF-36 Health-related Quality of Life Survey was used as a health outcome that represented a client-centred perspective. It has been reported that the SF-36 is not particularly responsive to modest improvements in hyperglycaemia^61^. However this broader health-related quality of life survey was chosen over diabetes-specific tools to capture the broader health implications of metabolic syndrome.

The primary limitation of this clinical trial was the sample size. Before commencement of the trial, power analysis estimated that 168 participants were required to detect a difference of 1% in HbA1c (α = 0.05, 90% power, *SD* = 1.4), allowing for a 20% noncompliance/withdrawal rate. The mean difference used for the power analysis was taken from the HbA1c smallest worthwhile clinical effect (also calculated as 1.5 mmol/L, *SD* = 1.0 for the FPG co-primary outcome measure). These were determined *a priori*, by clinical reasoning that considered the expense, harm, number of capsules, and the efficacy of alternative anti-hyperglycaemic medications.

The number of participants was restricted by slow recruitment. Metabolic syndrome is estimated to affect about one in four Australian adults. Therefore, we anticipated reasonable enrolment rates following initial screening. However many potential participants were found to have only two of the three necessary components of metabolic syndrome. The trial period was extended by 18 months, and the inclusion criteria were amended to allow enrolment of persons with non-alcoholic fatty liver syndrome. Upon trial completion, the sample size was 50% of that specified in the protocol. Although this trial provides valuable information regarding a metabolic syndrome population, the multiple-component definition of the disorder appears to present recruitment challenges for researchers.

There are additional considerations regarding whether the study is underpowered to determine efficacy of *Ganoderma lucidum* for reducing HbA1c. A *post hoc* power analysis suggested a smaller sample size might have been acceptable. Taking into account the 12% noncompliance and withdrawal rate observed here, and reducing the power to 80%, only 36 participants per group were required to detect a 1% difference in HbA1c at α = 0.05. Taken together with the pooling of the two *Ganoderma lucidum* intervention groups to improve statistical power, this suggests that the sample size might have been adequate for testing hypotheses for both primary outcomes. However, conventional power analyses are clearly arbitrary and conclusions on statistical significance remain extremely influenced by sample size^62^. Furthermore, statistical significance does not provide a magnitude of the effect size. For these reasons, clinical significance and interpretation of treatment effect sizes are increasingly regarded as the more important results from clinical trials.

The primary analysis provides the more important, statistically rigorous and clinically meaningful results of this trial. Secondary outcome measures might be underpowered to detect a significant difference between groups. Additionally, multiple analyses at multiple time points may introduce type 1 error. Here, the potential for this error was reduced by combining intervention groups and focussing on 16-week analyses only. However, no analyses in this trial produced a statistically significant result. The direction and magnitude of effects were examined for meaningful trends, but none were found. Similarly, the study was underpowered for inferential testing of safety variables and therefore these were evaluated with descriptive methods. Without a demonstration of even slight trends towards benefit, further research on *Ganoderma lucidum* for the treatment of cardiovascular risk factors of metabolic syndrome is difficult to justify.

There was no statistically significant or clinically significant effect of *Ganoderma lucidum* on hyperglycaemia, hypertension, or lipid profile in adults with metabolic syndrome or type 2 diabetes mellitus.

## Additional Information

**How to cite this article**: Klupp, N. L. *et al*. A double-blind, randomised, placebo-controlled trial of *Ganoderma lucidum* for the treatment of cardiovascular risk factors of metabolic syndrome. *Sci. Rep.* **6**, 29540; doi: 10.1038/srep29540 (2016).

## Supplementary Material

Supplementary Information

## Figures and Tables

**Table 1:** Daily dosage (mg) for each intervention with breakdown of components.

Intervention	Primary Components	Daily Dosage (mg)
*Ganoderma lucidum*	*Ganoderma lucidum* mushroom extract (10:1)	2,240
	*Ganoderma lucidum* spores	740
	Excipient	700
	Total	3,680
*Ganoderma lucidum* with *Cordyceps sinensis*	*Ganoderma lucidum* mushroom extract (10:1)	2,250
	*Ganoderma lucidum* spores	750
	*Cordyceps sinensis* mushroom extract	1,000
	Excipient	130
	Total	4,120
Placebo	Excipient	3,630

**Table 2:** Timing of outcome measures.

		Week 0	Week 4	Week 8	Week 12	Week 16	Week 24
		Outcome Measure		Baseline		Midpoint	Endpoint	Follow-up
Primary	HbA1c	✓		✓		✓	✓
	FPG	✓		✓		✓	✓
Secondary	Blood pressure	✓		✓		✓	✓
	Triglycerides	✓		✓		✓	✓
	Waist circumference	✓		✓		✓	✓
	Hip circumference	✓		✓		✓	✓
	BMI	✓		✓		✓	✓
	SF-36	✓		✓		✓	✓
	C-RP	✓		✓		✓	✓
	Total cholesterol	✓		✓		✓	✓
	HDL	✓		✓		✓	✓
	LDL	✓		✓		✓	✓
	Apolipoprotein A	✓				✓	
	Apolipoprotein B	✓				✓	

BMI = Body Mass Index, HDL-C = High Density Lipoprotein Cholesterol, C-RP = C-reactive protein, LDL = Low Density Lipoprotein Cholesterol.

**Table 3:** Participant demographic characteristics at baseline, for the combined intervention and placebo groups.

Demographics	Placebo (n = 30)	Combined Intervention Group (n = 54)	Test Statistic, *p*-value
Mean age, years (± SD)	57.1 (± 8.3)	60.2 (± 10.0)	*t*(82) = 1.44, *p* = 0.15
Sex, n (%)			
Male	17 (57)	27 (50)	*χ*^2^(1) = 0.34, *p* = 0.56
Female	13 (43)	27 (50)	
Country of birth, n (%)			
Australia	15 (50)	14 (52)	*χ*^2^(1) = 0.11, *p* = 0.75
Other than Australia	15 (50)	13 (48)	
Language, n (%)			
English	24 (80)	49 (91)	*χ*^2^(1) = 2.00, *p* = 0.16
Other than English	6 (20)	5 (9)	
Education, n (%)			
Postgraduate	4 (13)	8 (15)	*χ*^2^(3) = 0.07, *p* = 0.79
Undergraduate	5 (17)	12 (22)	
School	5 (17)	10 (19)	
Skills Training	16 (53)	24 (44)	
*Employment, n (%)			
Full-time	9 (30)	17 (31)	*χ*^2^(1) = 0.07, *p* = 0.79
Part-time	4 (13)	10 (19)	
Retired	9 (30)	20 (37)	
Unemployed	2 (7)	1 (2)	
Unable to work	2 (7)	4 (7)	
Home carer	4 (13)	2 (4)	
Carer of children under 18 years, n (%)			
Yes	7 (23)	8 (15)	*χ*^2^(1) = 0.95, *p* = 0.33
No	23 (77)	46 (85)	
Primary carer, n (%)			
Yes	2 (7)	5 (9)	*χ*^2^(1) = 0.17, *p* = 0.68
No	28 (93)	49 (91)	
Diabetes medication use, n (%)			
Yes	9 (30)	14 (26)	*χ*^2^(1) = 2.00, *p* = 0.16
No	21 (70)	40 (74)	

The appropriate test statistic and *p*-value are detailed in the right column.

^*^Due to small cell sizes, the working (full-time/part-time/caring) and not working (retired/unable to work/unemployed) groups were compared.

**Table 4:** Mean and SDs for each primary (reported to 2 dp) and secondary (reported to 1 dp) outcome measure at baseline and endpoint (16 weeks) for the combined *Ganoderma lucidum* and placebo groups.

		Placebo (n = 30) Mean (± *SD*)	Combined Intervention (n = 54) Mean (± *SD*)	Baseline-Adjusted Difference in Means, [95% CI]	*F*-value	*p*-value
Primary Outcome Measures						
HbA1c (%)	Baseline	7.40 (1.4)	7.69 (1.6)	0.13, [−0.35, 0.60]	*F*(1, 81) = 0.28	0.60
	16 weeks	7.29 (1.3)	7.66 (1.9)			
FPG (mmol/L)	Baseline	8.36 (2.7)	8.72 (2.9)	0.03, [−0.90, 0.96]	*F*(1, 81) = 0.00	0.95
	16 weeks	8.49 (2.6)	8.79 (3.2)			
Secondary Outcome Measures						
Arterial pressure (mmHg)	Baseline	99.6 (11.4)	100.9 (10.3)	−0.3, [−4.6, 3.9]	*F*(1, 81) = 0.02	0.88
	16 weeks	98.4 (10.7)	98.7 (11.4)			
Triglycerides (mmol/L)	Baseline	2.2 (0.9)	2.5 (2.8)	−0.1, [−0.3, 0.2]	*F*(1, 81) = 0.19	0.66
	16 weeks	2.2 (1.0)	2.6 (2.4)			
HDL (mmol/L)	Baseline	1.2 (0.2)	1.2 (0.5)	0.0 [−0.1, 0.1]	*F*(1, 81) = 0.02	0.90
	16 weeks	1.2 (0.2)	1.2 (0.4)			
Waist circumference (cm)	Baseline	113.3 (14.6)	109.6 (14.6)	0.7, [−1.5, 2.8]	*F*(1, 81) = 0.64	0.56
	16 weeks	113.3 (14.6)	110.5 (14.4)			
BMI (kg/m^2^)	Baseline	34.4 (7.4)	34.1 (6.8)	0.0, [−0.5, 0.5]	*F*(1, 81) = 0.00	0.99
	16 weeks	34.0 (7.3)	33.9 (6.6)			
SF-36 physical	Baseline	45.4 (8.2)	43.8 (8.8)	0.9, [−2.2, 4.0]	*F*(1, 81) = 0.31	0.58
	16 weeks	46.2 (8.5)	46.1 (8.9)			
SF-36 mental	Baseline	45.4 (8.2)	46.7 (13.5)	1.5, [−2.1, 5.0]	*F*(1, 81) = 0.65	0.42
	16 weeks	48.5 (12.7)	48.5 (11.1)			
C-reactive protein (mg/L)	Baseline	3.1 (2.9)	4.8 (6.3)	−0.7, [−2.8, 1.4]	*F*(1, 74) = 0.41	0.53
	16 weeks	4.1 (6.3)	3.9 (3.5)			
Total cholesterol (mmol/L)	Baseline	5.2 (1.2)	4.9 (1.3)	0.2, [−0.2, 0.5]	*F*(1, 81) = 0.73	0.39
	16 weeks	5.1 (1.2)	5.0 (1.5)			
LDL (mmol/L)	Baseline	2.9 (1.1)	2.7 (1.0)	0.2, [−0.1, 0.5]	*F*(1, 80) = 1.48	0.23
	16 weeks	2.8 (1.0)	2.8 (1.2)			
Apolipoprotein A (g/L)	Baseline	1.4 (0.3)	1.4 (0.3)	0.0, [−0.1, 0.1]	*F*(1, 72) = 0.01	0.90
	16 weeks	1.4 (0.3)	1.4 (0.3)			
Apolipoprotein B (g/L)	Baseline	0.9 (0.3)	0.9 (0.2)	0.0, [−0.1, 0.1]	*F*(1, 72) = 0.00	0.98
	16 weeks	0.9 (0.3)	0.9 (0.3)			

Baseline-adjusted difference in means, 95% CI, and *F*- and *p*-values are also reported. For C-reactive protein, LDL cholesterol, and apolipoproteins A and B, there were missing data at baseline and LOCF could not be carried out, hence the degrees of freedom differ for these outcomes.
