# Supplementary material for: A double-blind, randomised, placebo-controlled trial of Ganoderma lucidum for the treatment of cardiovascular risk factors of metabolic syndrome
Source: Sci Rep. 2016 Aug 11;6:29540. doi: 10.1038/srep29540 (PMC4980683; doi:10.1038/srep29540)
Supplement: Supplementary Information [file srep29540-s1.pdf]

SUPPLEMENTARY MATERIALS

Manuscript Title: A double-blind, randomised, placebo-controlled trial of *Ganoderma lucidum* for the treatment of cardiovascular risk factors of metabolic syndrome

Authors: Nerida L. Klupp, Hosen Kiat, Alan Bensoussan, Genevieve Z. Steiner, Dennis H. Chang

Week Withdrawn	Allocated Group	Reason for Withdrawal
Baseline	Placebo	General practitioner prescribed insulin to manage hyperglycaemia
Week 1	*Ganoderma lucidum* with *Cordyceps sinensis*	Participant had stroke*
Week 1	*Ganoderma lucidum* with *Cordyceps sinensis*	General practitioner prescribed insulin to manage hyperglycaemia
Week 1	*Ganoderma lucidum*	Participant was concerned about erratic blood glucose levels and other illness priorities and changed his/her mind
Week 4	Placebo	Participant required thyroidectomy after enlarged thyroid was detected during screening by trial physician
Week 4	*Ganoderma lucidum* with *Cordyceps sinensis*	Participant was concerned that high blood glucose levels might be caused by trial medication
Week 12	Placebo	Participant was diagnosed with breast cancer*
Week 12	*Ganoderma lucidum*	Participant developed urinary tract infection requiring antibiotics and did not want to take so many tablets
Week 16	Placebo	Participant was not interested in final follow-up appointment

*Reported to TGA for serious adverse events.

Supplementary Materials Table 1. The week participants withdrew from the study (left), the intervention group that they had been randomly allocated to (middle), and their reasons for withdrawal (right).

	Ganoderma lucidum (n = 27)		Placebo (n = 30)		*Ganoderma lucidum* with *Cordyceps sinensis* (n = 27)	
	Week 0-16	Week 16-24	Week 0-16	Week 16-24	Week 0-16	Week 16-24
**Anticipated adverse events**						
Headache	1		1		2	
Fatigue	6	1	1		1	3
Diarrhoea	6		4		4	
Constipation	3		3			
Nausea	4	1	1		4	
**Nonanticipated adverse events**						
Immune system and infection	9	1	14	5	11	1
Skin	1		4		1	1
Neurological	2	1			1	
Musculoskeletal	6		4			
Liver and biliary						
Alimentary and pancreatic	5		5	1	3	1
Endocrine	2		1		3	
Respiratory	5	3	5		3	
Cardiovascular		1	3			
Kidney and genitourinary						1
Mental health	2		2	2	2	3
**Total**	**52**	**8**	**48**	**8**	**35**	**10**

Supplementary Materials Table 2. The frequency of anticipated mild adverse events (side effects) and non-anticipated adverse events separately for the 3 intervention groups from the intervention (0-16 Weeks) and follow-up (16-24 Weeks) periods.
